# Coherent Control of Molecular Dissociation by Selective Excitation of Nuclear Wave Packets

**DOI:** 10.3389/fchem.2022.859095

**Published:** 2022-04-05

**Authors:** Hugo A. López Peña, Jacob M. Shusterman, Derrick Ampadu Boateng, Ka Un Lao, Katharine Moore Tibbetts

**Affiliations:** Department of Chemistry, Virginia Commonwealth University, Richmond, VA, United States

**Keywords:** coherent control, strong field ionization, mass spectrometry, pump-probe, nuclear wave packet

## Abstract

We report on pump-probe control schemes to manipulate fragmentation product yields in *p*-nitrotoluene (PNT) cation. Strong field ionization of PNT prepares the parent cation in the ground electronic state, with coherent vibrational excitation along two normal modes: the C–C–N–O torsional mode at 80 cm^−1^ and the in-plane ring-stretching mode at 650 cm^−1^. Both vibrational wave packets are observed as oscillations in parent and fragment ion yields in the mass spectrum upon optical excitation. Excitation with 650 nm selectively fragments the PNT cation into ${\text{C}}_{7}{{\text{H}}_{7}}^{+}$ , whereas excitation with 400 nm selectively produces ${\text{C}}_{5}{{\text{H}}_{5}}^{+}$ and ${\text{C}}_{3}{{\text{H}}_{3}}^{+}$ . In both cases the ion yield oscillations result from torsional wave packet excitation, but 650 and 400 nm excitation produce oscillations with opposite phases. Ab initio calculations of the ground and excited electronic potential energy surfaces of PNT cation along the C–C–N–O dihedral angle reveal that 400 nm excitation accesses an allowed transition from D_0_ to D_6_ at 0° dihedral angle, whereas 650 nm excitation accesses a strongly allowed transition from D_0_ to D_4_ at a dihedral angle of 90°. This ability to access different electronic excited states at different locations along the potential energy surface accounts for the selective fragmentation observed with different probe wavelengths. The ring-stretching mode, only observed using 800 nm excitation, is attributed to a D_0_ to D_2_ transition at a geometry with 90° dihedral angle and elongated C–N bond length. Collectively, these results demonstrate that strong field ionization induces multimode coherent excitation and that the vibrational wave packets can be excited with specific photon energies at different points on their potential energy surfaces to induce selective fragmentation.

## 1 Introduction

Chemists have sought to control molecular dissociation with lasers for decades. Tunable monochromatic laser light was believed to enable “bond-selective chemistry” through resonant energy absorption at the vibrational frequency of the targeted bond Bloembergen and Yablanovitch (1978). However, early attempts to control bond-cleavage by tuning the laser frequency failed due to rapid intramolecular vibrational energy redistribution (IVR) across coupled vibrational modes Bloembergen and Zewail (1984). Effective control of unimolecular dissociation was only achieved with the development of high-intensity ultrashort pulsed lasers and coherent control techniques that operate on timescales faster than IVR.

Coherent control over molecular dissociation has primarily been achieved by the “closed-loop” scheme of optimally designing shaped laser pulses with automated learning algorithms, initially proposed by Judson and Rabitz (1992). Gerber and co-workers reported the first experimental implementation of closed-loop control over ionization and dissociation of CpFe(CO)_2_Cl (Cp = cyclopentadienyl) into CpFeCOCl^+^ or FeCl^+^ Assion et al. (1998). This success spurred the application of closed-loop control to selectively dissociate various molecules including halogenated alkanes Damrauer et al. (2002), Langhojer et al. (2005), Plenge et al. (2011), Moore Tibbetts et al. (2013) and acetones Langhojer et al. (2005), Cardoza et al. (2006). However, shaped pulse control fails to enhance specific dissociation pathways in certain molecules including *p*-nitrotoluene Lozovoy et al. (2008). Moreover, the “black box” nature of closed-loop control makes it difficult to fully understand the physical mechanisms by which an optimal pulse shape achieves product selectivity, even using additional specialized pulse shaping procedures Xing et al. (2017).

Understanding the physical mechanisms underlying coherent control of molecular dissociation can be achieved using two-pulse “pump-probe” excitation schemes Tannor and Rice (1985), Zewail (1988). Pump-probe measurements with complementary quantum chemical calculations of the relevant electronic potential energy surfaces (PESs) have revealed bond-cleavage mechanisms facilitated by coherent vibrational motions in numerous organic cations Moore Tibbetts (2019). For instance, coherent excitation of the I–C–Br bending mode in CH_2_IBr^+^ upon strong-field ionization facilitates dissociation into CH_2_Br^+^ upon excitation of the D_0_→D_3_ transition at a specific point on the D_0_ PES Nichols et al. (2009). Similarly, coherent vibrational motions along the phenyl–substituent torsional coordinate in the molecular cations of acetophenone Bohinski et al. (2014), Tibbetts et al. (2015) and nitrobenzene López Peña et al. (2021) facilitate CH_3_ and NO_2_ loss, respectively, upon excitation of the vibrational wave packet at 90° phenyl–substituent dihedral angle. Although pump-probe measurements advance understanding of molecular dissociation facilitated by coherent vibrational dynamics, they enable only limited control over relative fragment yields because the probe wavelength typically excites resonantly to a single electronic excited state. As a result, one preferential fragment or a specific distribution of fragments is usually observed.

In this work, we demonstrate selective coherent excitation to three different electronic excited states from ground-state *p*-nitrotoluene (PNT) cation using probe pulses at 800, 650, and 400 nm. This selective excitation results in different relative yields of the ${\text{C}}_{7}{{\text{H}}_{7}}^{+}$ , ${\text{C}}_{5}{{\text{H}}_{5}}^{+}$ , and ${\text{C}}_{3}{{\text{H}}_{3}}^{+}$ fragment ions depending on the probe wavelength. Strong field adiabatic ionization prepares a superposition of two vibrational wave packets in the D_0_ PES of PNT cation: the first along the C–C–N–O torsional coordinate identified in earlier work Ampadu Boateng et al. (2018) and the second along the in-plane phenyl ring-stretching mode that includes C–N bond stretching. The torsional wave packet can be selectively excited to D_4_ at a C–C–N–O dihedral angle of 90° with 650 nm photons to produce primarily ${\text{C}}_{7}{{\text{H}}_{7}}^{+}$ , or to D_6_ at a 0° dihedral angle with 400 nm photons to produce ${\text{C}}_{5}{{\text{H}}_{5}}^{+}$ and ${\text{C}}_{3}{{\text{H}}_{3}}^{+}$ . The ring-stretching wave packet can be selectively excited to D_2_ at a geometry with slightly elongated C–N bond length and 90° dihedral angle using 800 nm photons, producing exclusively ${\text{C}}_{7}{{\text{H}}_{7}}^{+}$ . These results indicate that careful choice of excitation wavelengths in two-pulse schemes can effectively control dissociation pathways in a complex organic molecule.

## 2 Materials and Methods

### 2.1 Experiments

Portions of the experimental setup have been described in our previous works Ampadu Boateng et al. (2018), Ampadu Boateng et al. (2019a). Briefly, a commercial Ti:sapphire regenerative amplifier (Astrella, Coherent, Inc.) producing 30 fs, 800 nm, 2.2 mJ pulses was used to pump an optical parametric amplifier (OPA, TOPAS Prime) to produce sub-20 fs 1,300 nm or 1,500 nm pump pulses. The pump wavelength was 1,300 nm for measurements with the 400 and 650 nm probes, whereas the pump wavelength was 1,500 nm for measurements with the 800 nm probe for reasons that will be discussed in the Results section. The 650 nm probe pulse was obtained from OPA output split with a 50:50 (r:t) beam splitter and frequency doubled with a *β*-barium borate (BBO) crystal. The 800 nm probe pulse was obtained from the transmitted portion of the incident laser beam from a 90:10 (r:t) beam splitter prior to the OPA and down-collimated using a reflective telescope with reduction factor 2. The 400 nm probe pulse was obtained by down-collimating the 800 nm beam with a telescope with reduction factor 3.33 comprised of a plano-convex lens (*f* = 250 mm) and a plano-concave lens (*f* = −75 mm) placed on a linear translation stage, followed by frequency doubling with a BBO crystal. The transmissive telescope geometry was necessary to allow for fine adjustment of the focal spot of the 400 nm probe beam to overlap with the focal spot of the 1,300 nm pump beam when both are focused with the same plano-convex lens (*f* = 200 mm). Both the 800 and 650 nm probe pulses have duration of ∼25 fs as measured by frequency-resolved optical gating Ampadu Boateng et al. (2018), Ampadu Boateng et al. (2019a). The 400 nm probe pulse duration was estimated at 70 fs as measured by the cross-correlation of the O_2_ signal from air in the mass spectrometer with 1,300 nm pump/400 nm probe excitation (Supplementary Figure S1). PNT (Sigma Aldrich, 99%) was introduced into the time-of-flight mass spectrometer (Jordan TOF) *via* an effusive inlet under gentle heating. Pump-probe measurements were taken over the delay range of −500 fs (probe before pump) to +2,500 fs (pump before probe) in steps of 5 fs for the 800 nm probe and 10 fs otherwise. Mass spectra were recorded at each pump-probe delay and averaged over 1,000 laser shots with a 1 GHz digital oscilloscope (LeCroy WaveRunner 610Zi).

### 2.2 Computations

Density functional theory (DFT) calculations were conducted using Gaussian 16 software Frisch et al. (2016) employing the restricted Kohn-Sham formalism for neutral species and the unrestricted formalism for cationic species. A previous work on PNT from our group Ampadu Boateng et al. (2018a) has identified the hybrid generalized gradient approximation (GGA) B3LYP functional Becke (1993), Stephens et al. (1994) in combination with the def2-TZVPP Weigend and Ahlrichs (2005) basis set as an adequate level of theory to describe this molecular system. Both the neutral and cation geometries of PNT were optimized within this level of theory. The convergence threshold for total energy was set to 10^–8^ eV while the force threshold was set to 10^–3^ eV/Å. Each geometric optimization was followed by harmonic frequency computations in order to confirm the stationary character of the state obtained.

To determine the excited-state energies of the PNT cation at different geometries, we performed single-point time-dependent DFT (TDDFT) Bauernschmitt and Ahlrichs (1996) calculations using Gaussian 16. For each cation geometry, we calculated the first 10 doublet-doublet transitions at the B3LYP/def2-TZVPP level of theory. In section 3.2 we will present TDDFT excited-state calculations for PNT radical cation at the B3LYP/def2-TZVPP level but those calculations will be further supported with selected calculations at the equation-of-motion excitation-energies coupled-cluster singles and doubles (EOM-EE-CCSD) Krylov, (2008) level. Due to the high computational cost of EOM methods, we employ the smaller 6−311+G* basis set. These EOM calculations were performed using Q-Chem 5.3 Epifanovsky et al. (2021). It is important to clarify that both TDDFT and EOM calculations on the cation are done under field-free conditions, i.e., after the strong-field pump pulse is over (see further discussion on Section 3.1).

## 3 Results

### 3.1 Pump-Probe Measurements

**FIGURE 1:**
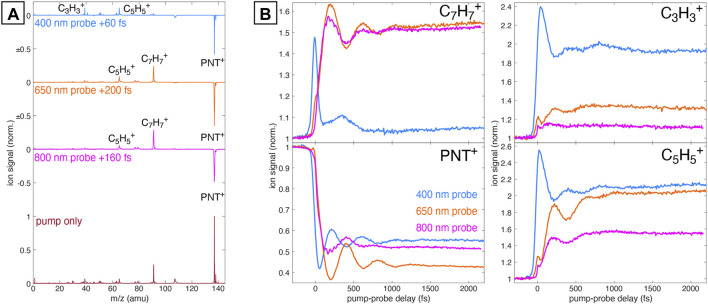
**(A)** Mass spectrum of PNT^+^ taken with 1,300 nm pump only (dark red) and the difference spectra relative to the pump-only spectrum taken with 800 nm (magenta), 650 nm (orange), and 400 nm (blue) probe pulses at the indicated delays. **(B)** Transient ion signals for PNT^+^, ${\text{C}}_{7}{{\text{H}}_{7}}^{+}$ , ${\text{C}}_{5}{{\text{H}}_{5}}^{+}$ , and ${\text{C}}_{3}{{\text{H}}_{3}}^{+}$ taken at each probe wavelength. Each signal is normalized to its yield at negative pump-probe delay.

Pump-probe measurements were conducted with pump intensity of 6 × 10^13^ W cm^−2^. Figure 1A shows the mass spectra of PNT^+^ taken with only the 1,300 nm pump pulse (bottom) and with pump-probe excitation using 6 × 10^12^ W cm^−2^ probe pulses at 800, 650, and 400 nm (top). The pump-only spectrum is dominated by the intact PNT^+^ cation at *m*/*z* 137, with minor contribution from the ${\text{C}}_{7}{{\text{H}}_{7}}^{+}$ fragment at *m*/*z* 91. The greatest depletion in PNT^+^ signal was observed at pump-probe delays of +160 fs for 800 nm probe, +200 fs for 650 nm probe, and +60 fs for 400 nm probe. As seen in Figure 1A, a substantial depletion in PNT^+^ signal and concomitant rise in fragment ion signals occurs at the selected pump-probe delay for each probe wavelength. Whereas both the 800 and 650 nm probe wavelengths primarily enhance the yield of ${\text{C}}_{7}{{\text{H}}_{7}}^{+}$ and to a lesser extent ${\text{C}}_{5}{{\text{H}}_{5}}^{+}$ (*m*/*z* 65), the 400 nm probe enhances only ${\text{C}}_{5}{{\text{H}}_{5}}^{+}$ and ${\text{C}}_{3}{{\text{H}}_{3}}^{+}$ (*m*/*z* 39). This change in fragmentation pattern with different probe wavelengths indicates that selective fragmentation is possible using pump-probe excitation.

Figure 1B displays the transient ion signals of the PNT^+^, ${\text{C}}_{7}{{\text{H}}_{7}}^{+}$ , ${\text{C}}_{5}{{\text{H}}_{5}}^{+}$ , and ${\text{C}}_{3}{{\text{H}}_{3}}^{+}$ fragments as a function of pump-probe delay using 800 nm (magenta), 650 nm (orange), and 400 nm (blue) probe wavelengths. Each ion signal is normalized to its respective yield at negative pump-probe delay. The large-amplitude oscillations in the PNT^+^ and fragment ion signals with period 420 fs arise from the vibrational wave packet along the C–C–N–O torsional coordinate in PNT^+^, which we previously reported from pump-probe measurements with only 800 nm probe wavelength Ampadu Boateng et al. (2018a). The present results show that the 650 nm probe wavelength produces larger-amplitude oscillations in PNT^+^ with the same phase, which indicates that 650 nm more effectively excites the PNT^+^ torsional wave packet than 800 nm. In contrast, the PNT^+^ oscillations with the 400 nm probe have the opposite phase, which indicates 400 nm probe selectively excites the PNT^+^ torsional wave packet at a different location on the PES along the torsional coordinate than the lower-energy probe wavelengths. Finally, additional low-amplitude fast oscillations with a period of ∼55 fs are observed in the PNT^+^ and ${\text{C}}_{7}{{\text{H}}_{7}}^{+}$ signals only for the 800 nm probe. These oscillations were best resolved using a 1,500 nm pump wavelength (shown in Figure 1B), although they are also visible using a 1,300 nm pump wavelength (Supplementary Figure S2).

To further interpret the oscillatory dynamics, transient ion signals as a function of pump-probe delay, *τ*, were fit to the equation

$$S\left(\tau \right)=ae^{-\tau /T_{1}}\cos\left(\frac{2\pi }{t}\tau +\phi \right)+be^{-\tau /T_{2}}+c$$ (1)

where *a* and *b* are amplitude coefficients, *t* is the oscillation period, *T*_1_ is the coherence lifetime, *T*_2_ is a second lifetime not associated with oscillations, and *c* is the ion yield as *τ*→*∞*. Each transient signal at *τ* ≥ 70 fs (i.e., after the pump pulse is over so the instrument response function can be ignored) was fit to Eq. 1 using nonlinear least-squares curve fitting in MATLAB. A full description of the extracted coefficients can be found in the Supplementary Material, Supplementary Figure S3 and Supplementary Tables S1–S3. Although the strong-field pump pulse can populate multiple electronic states of the cation, it is reasonable to assume that, when the probe beam arrives after a delay ≥70 fs, it will find the cation in the ground electronic state after electronic relaxation. The validity of this assertion can be supported with the work of Kraus and coworkers Kraus et al. (2015), in which charge migration processes leading to relaxation of the electronic wave packet in iodoacetylene occur within 5 fs, well below the initial measurement of dynamics at 70 fs delay used for our analysis. The fit coefficients corresponding to the oscillatory dynamics shown in Table 1 confirm both that each fragment ion oscillates *π* radians out of phase with respect to PNT^+^ and that the phase of the PNT^+^ signal shifts from approximately 0 radians for 650 nm excitation to *π* radians for 400 nm excitation. The oscillation period is ∼420 fs for both 650 and 400 nm probes, whereas the somewhat longer ∼460 fs oscillation period using the 800 nm probe arises from a poorer fit quality (Supplementary Figure S3).

**TABLE 1:** Curve fitting coefficients for coherent dynamics of ion signals: oscillation amplitude (*a*), coherent lifetime (*T*_1_), oscillation period (*t*), and phase (*ϕ*).

*λ*_*probe*_ (nm)	Ion	*a*	*T*_1_ (fs)	*t* (fs)	*ϕ* (rad)
400	PNT^+^	0.10 ± 0.01	345 ± 33	410 ± 7	3.1 ± 0.1
—	${\text{C}}_{5}{{\text{H}}_{5}}^{+}$	0.03 ± 0.01	370 ± 120	421 ± 20	0.3 ± 0.3
650	PNT^+^	0.25 ± 0.01	308 ± 15	425 ± 4	0.05 ± 0.04
—	${\text{C}}_{7}{{\text{H}}_{7}}^{+}$	0.15 ± 0.01	290 ± 12	417 ± 3	3.1 ± 0.03
—	${\text{C}}_{5}{{\text{H}}_{5}}^{+}$	0.09 ± 0.01	252 ± 17	438 ± 9	3.3 ± 0.1
800	PNT^+^	0.15 ± 0.02	210 ± 16	464 ± 11	0.45 ± 0.07
—	${\text{C}}_{7}{{\text{H}}_{7}}^{+}$	0.13 ± 0.01	212 ± 12	438 ± 7	3.3 ± 0.1

**FIGURE 2:**
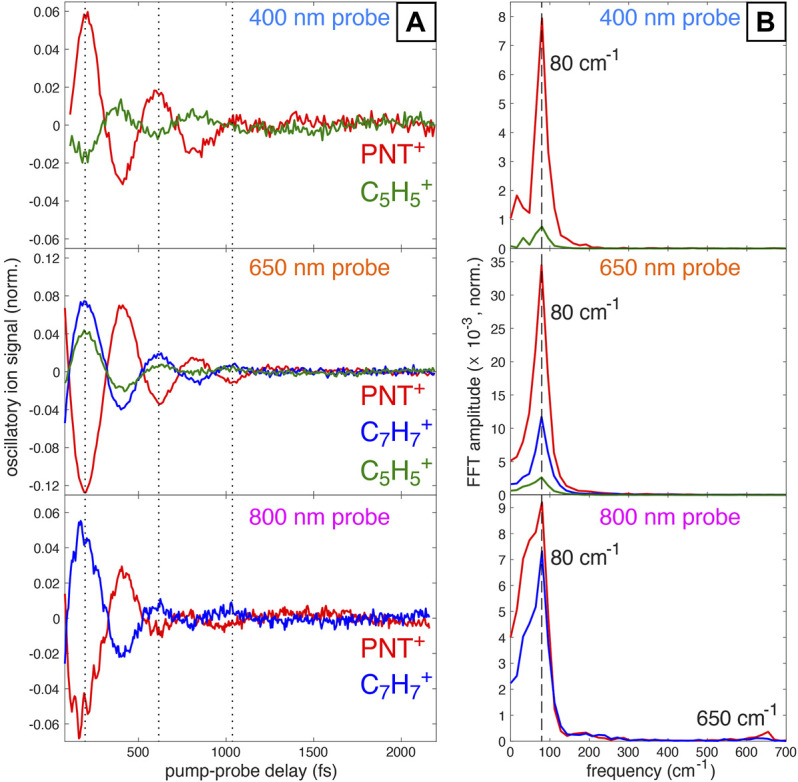
**(A)** Oscillatory ion signals for PNT^+^ and selected fragment ions obtained by subtracting off the incoherent contributions to signal fitting *via* Eq. 1. **(B)** FFT amplitudes obtained from the signals in **(A)** with indicated frequencies at 80 cm^−1^ and 650 cm^−1^ (for 800 nm probe).

Subtraction of the incoherent dynamics (second and third terms in Eq. 1) allows for clearer visualization of the oscillatory dynamics and frequency analysis via fast Fourier Transform (FFT). Figure 2A displays the coherent transient ion dynamics of PNT^+^ and oscillatory fragment ions for each probe wavelength. A clear *π* phase shift in the PNT^+^ signals (red) between the 400 nm probe and 650 nm or 800 nm probes is visible, as indicated by the dotted lines at 200, 620, and 1,040 fs. Figure 2B displays the FFT amplitude of the signals shown in Figure 2A. For all probe wavelengths the FFT spectra exhibit a strong peak at 80 cm^−1^ assigned to the previously reported C–N–N–O torsional mode of PNT^+^ Ampadu Boateng et al. (2018). A closer inspection of the oscillations using the 800 nm probe (bottom panel of Figure 2A) reveals what seems to be the superposition of two coherent oscillations: one corresponding to the torsional mode already discussed and smaller amplitude oscillations corresponding to a faster vibrational mode. In line with these observations is the presence of two frequencies, at 80 and 650 cm^−1^, in the FFT of the 800 nm probe signal (bottom panel of Figure 2B). Additionally, it is worth noting that the superposition of vibrational modes is more evident around the first minimum of the oscillatory ion signal in PNT^+^. This fact will be further discussed in Section 3.3 with the aid of computational results.

**FIGURE 3:**
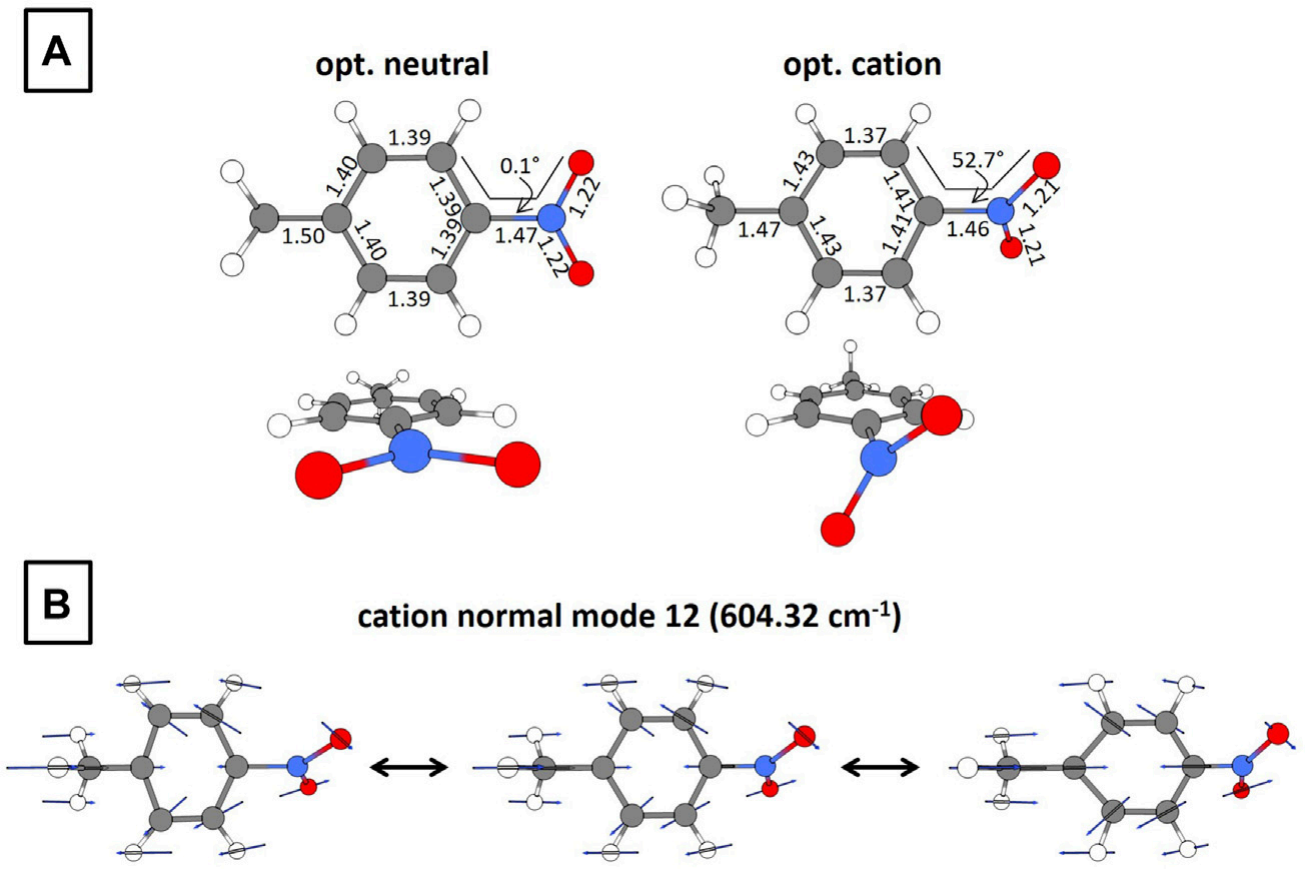
**(A)** Geometrical structures for optimized neutral and cationic PNT. Bond lengths are in Å and torsional angles in degrees. **(B)** Normal mode at 604.32 cm^−1^ for the optimized PNT cation. The calculations were performed at the B3LYP/def2-TZVPP level of theory.

In order to motivate forthcoming computational results, we present the optimized structures of neutral and cationic PNT at the B3LYP/def2-TZVPP level of theory in Figure 3. The corresponding coordinates are available within the Supplementary Material (Supplementary Tables S4, S5). Relevant to this work are the following changes after electron detachment: the C–C–N–O dihedral angle goes from 0.1 to 52.7° and a moderate distortion of the ring occurs. Additionally, the frequencies and corresponding intensities of the normal modes for PNT^+^ are presented in the Supplementary Material (Supplementary Table S6). A careful analysis of these vibrational modes shows that the C–C–N–O torsional mode has a calculated frequency of 58.96 cm^−1^, in reasonable agreement with the experimental frequency of 80 cm^−1^ (Figure 2B). Figure 3B shows the 12th normal mode calculated for the PNT cation. The relevance of this ring-stretching mode will be further discussed in Section 3.3. At this moment, it is enough to say that the calculated frequency of 604.32 cm^−1^ fairly matches the experimental frequency of 650 cm^−1^ shown in Figure 2B for the 800 nm probe.

### 3.2 Coherent Control of Torsional Wave Packet Excitation

**FIGURE 4:**
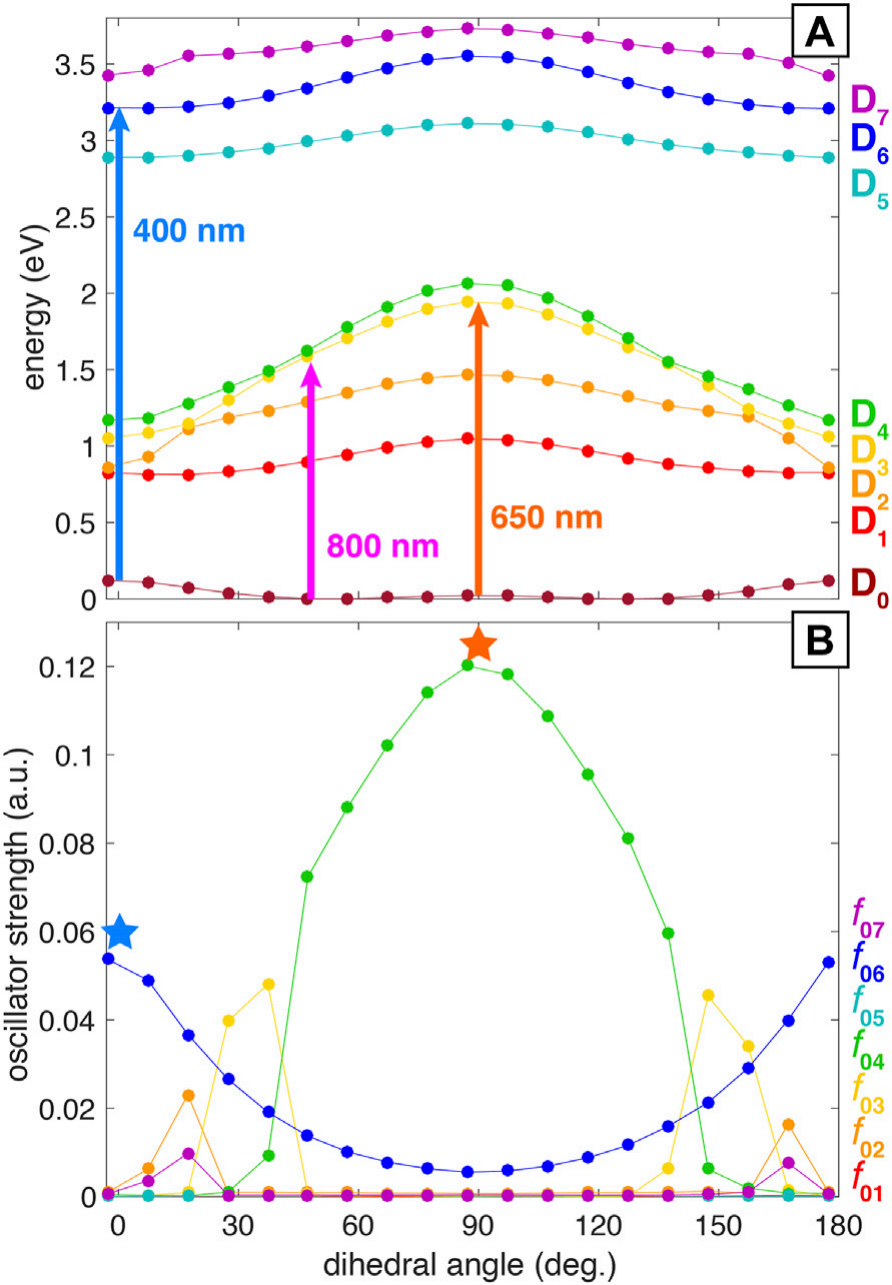
Computed potential energy surfaces **(A)** and oscillator strengths **(B)** for PNT^+^ along the C–C–N–O dihedral angle.

As mentioned in the previous section, a former work from our group has assigned the main coherent oscillations of PNT^+^ to a torsional wave packet involving the C–C–N–O dihedral angle Ampadu Boateng et al. (2018). In order to explain the observation of different fragment distributions and ion yield dynamics depending on the wavelength of the probe beam, we used the following approach: starting from the optimized cation geometry with a C–C–N–O dihedral angle of 52.7° we performed a relaxed scan of this torsional mode using the ModRedundant keyword in Gaussian 16. This procedure generated a collection of geometries that span all the torsional mode, which were used to perform single-point TDDFT calculations. The resulting potential energy surfaces (PESs) can be seen in Figure 4A. In line with previous findings on the closely related nitrobenzene cation López Peña et al. (2021), we found that the PESs can be classified into two groups: the first one comprising from D_1_ to D_4_ with all the surfaces showing a marked dependence on the dihedral angle, and a second group comprising from D_5_ to D_7_ with a less marked dependence on the dihedral angle. The influence of the dihedral angle on the excitation probability, quantified by means of a harmonic estimate of the oscillator strength, can be observed in Figure 4B. It is noteworthy that the oscillator strength for the D_0_→D_4_ transition (*f*_04_) is particularly high and dependent on the geometry, reaching its maximum value at 90°. Moreover, this transition is the only one that has a substantial oscillator strength at 90°. Also notable is the parabolic shape of *f*_06_ centered at 90° dihedral angle. As a consequence, there is higher probability for the D_0_→D_6_ transition at geometries where PNT^+^ is nearly planar, i.e., near the neutral PNT geometry. Figure 4A also shows the energies corresponding to 400, 650, and 800 nm photons for comparison (3.1, 1.91, and 1.55 eV respectively). A careful evaluation of all the information contained in Figure 4 as a whole reveals that the 400 nm probe can selectively access an allowed transition from D_0_ to D_6_ at dihedral angles close to 0°, whereas the 650 nm probe can selectively access a strongly allowed transition from D_0_ to D_4_ at a dihedral angle of 90°. This ability to access different electronic excited states at different locations along the potential energy surface accounts for the selective fragmentation observed with different probe wavelengths at different time delays. Additionally, the 800 nm probe should be capable of promoting the D_0_→D_*n*_, *n* = 1, 2 transitions at any dihedral angle.

In order to support our TDDFT calculations we also performed single-point computations at three selected geometries calculated at the B3LYP/def2-TZVPP level using the EOM-EE-CCSD method. The geometries considered were the optimized neutral and cationic species (Figure 3A; Supplementary Tables S4, S5) and the geometry with a dihedral angle of 87.4° obtained by means of the relaxed scan previously described (Supplementary Table S7). Table 2 shows the excitation energies and harmonic estimates of oscillator strengths calculated at both levels of theory for PNT cation with a C–C–N–O dihedral angle of 87.4°. These methodologies produced qualitatively similar results but there are some differences that are worth noting: EOM calculations introduce a larger energetic gap between D_1_ and D_2_ states of ∼1.2 eV. Consequently, states D_2_, D_3_, and D_4_ from EOM computations are shifted upwards when compared with TDDFT results. Also, according to the oscillator strength values, EOM method points towards D_3_ as the bright state while TDDFT locates the D_4_ state as the bright one. Despite this disagreement in the ordering of states, the relevant fact is that both methodologies confirm the presence of an excited state with substantial oscillator strength at 90° dihedral angle and with excitation energy around 2 eV. Possible sources for the disagreement between both levels of theory will be briefly discussed on Section 4. Supplementary Tables S8, S9 of Supplementary Material show analogous comparisons between the two methodologies for PNT^+^ at the optimized neutral and cation geometries respectively.

**TABLE 2:** Excitation energies (EE) and oscillator strengths (*f*) for PNT^+^ with a C–C–N–O dihedral angle of 87.4° at the B3LYP/def2-TZVPP and EOM-EE-CCSD/6-311+G* levels of theory.

	B3LYP/		EOM-EE-CCSD/	
	def2-TZVPP		6−311 + G*	
Transition	EE (eV)	*f* (a.u.)	EE (eV)	*f* (a.u.)
D_0_→D_1_	1.02	0.0000	1.12	0.000049
D_0_→D_2_	1.44	0.0007	2.35	0.000654
D_0_→D_3_	1.92	0.0000	2.59	0.132253
D_0_→D_4_	2.04	0.1201	2.87	0.000112
D_0_→D_5_	3.09	0.0001	3.49	0.000003
D_0_→D_6_	3.53	0.0055	3.56	0.000051
D_0_→D_7_	3.71	0.0004	3.84	0.001330

### 3.3 Assignment of 650 cm^−1^ Wave Packet

In Section 3.1 we showed experimental evidence of the superposition of two vibrational wave packets and identified the associated frequencies as 80 and 650 cm^−1^. The 80 cm^−1^ has previously been assigned to the C–C–N–O torsional mode but the identity of the vibrational mode associated with the 650 cm^−1^ frequency remains to be fully elucidated. The match between the experimental frequency of 650 cm^−1^ (Figure 2B) and the computationally calculated frequency of 604.32 cm^−1^ (Figure 3B and Supplementary Table S6) lays the foundation for the hypothesis that this ring-stretching mode is the one supporting the additional nuclear wave packet found in this work.

In order to test this hypothesis we simulated the superposition of the two vibrational modes by considering three geometries within the torsional mode with 0.1, 52.7, and 87.4 C–C–N–O dihedral angles. Then, from these three geometries we performed frequency calculations to identify the ring-stretching mode in each case. The geometry with 0.1° torsional angle corresponds to the vertical cation, i.e., the cation under the optimized neutral geometry, while the structure with 52.7° dihedral angle corresponds to the optimized cation with a ring-stretching mode at 604.32 cm^−1^ (Supplementary Table S6). Having identified the ring-stretching modes for each of the three geometries we made the additional hypothesis that the D_0_→D_2_ transition is the one allowing the observation of the ring-stretching wave packet. This hypothesis is supported by our TDDFT calculations showing that 800 nm is nearly resonant with the D_0_→D_2_ transition at any dihedral angle (Figure 4A) and by the experimental fact that the superposition of vibrational wave packets is only observable with 800 nm excitation. With these ideas in mind we took five “snapshots” within each of the three ring-stretching modes and retrieve the corresponding geometries. Those 15 geometries, five per each ring-stretching mode, served as the basis for single-point TDDFT calculations to obtain the D_0_→D_2_ excitation energies and the corresponding harmonic estimates for the oscillator strengths.

**FIGURE 5:**
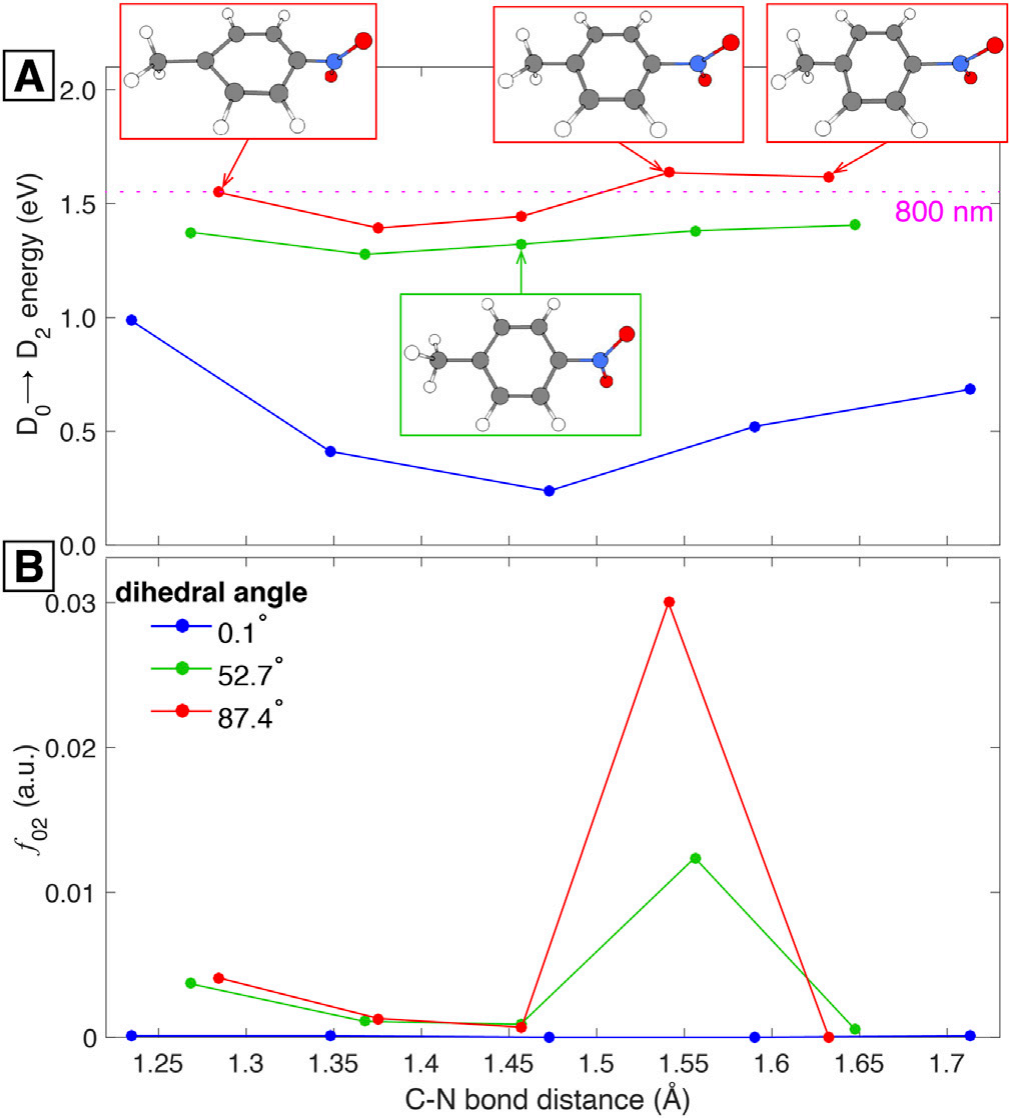
Computed potential energy surfaces **(A)** and oscillator strengths **(B)** for PNT^+^ along C–N bond distance under different C–C–N–O dihedral angles.

The results of these calculations are shown in Figure 5. It is important to offer some clarification regarding the abscissa axis of this figure: since the ring-stretching mode involves the collective motion of many atoms as can be seen in Figure 3B, the description of the mode in terms of a single parameter is not an easy task. Due to this complication we show the C–N bond distance as a signature of the mode, but it should be kept in mind that the abscissa axis represents the whole ring-stretching mode, as highlighted in the structures shown in Figure 5. This being said, Figure 5A shows the excitation energy for the D_0_→D_2_ transition as a function of the C–N bond distance for three different torsional geometries with 0.1, 52.7, and 87.4 dihedral angles. The figure also shows that 800 nm (1.55 eV) provides enough energy to promote the D_0_→D_2_ transition for all the C–N bond lengths under all torsional geometries. Additionally, 800 nm is nearly resonant with the excitation energy corresponding to the torsional geometry of 87.4° at all C–N bond distances. This last observation might explain why the superposition of vibrational modes is more evident around the first minimum of PNT^+^ and the first maximum of ${\text{C}}_{7}{{\text{H}}_{7}}^{+}$ oscillatory ion signals (bottom panel of Figure 2A). The reasons behind the association of the minimum in the oscillatory ion signal of PNT^+^ with a dihedral angle ∼90° are two-fold: first, our TDDFT calculations show that there is a strong probability of a D_0_→D_4_ transition as the dihedral angle approaches 90° (Figure 4). While it is true that 800 nm excitation is not capable of promoting the D_0_→D_4_ at all dihedral angles it is also true that it can promote such transition at different torsional angles. The second reason is motivated by a previous work from our group regarding an analogous torsional wave packet within nitrobenzene cation, a cation closely related to PNT^+^. In that work we estimated the time taken for the wave packet to reach a 90° dihedral angle as approximately 200 fs by means of classical wave packet trajectory calculations and pump-probe measurements. This finding is consistent with the maximum depletion of PNT^+^ at 160 fs pump-probe delay with 800 nm excitation (the maximum depletion with 650 nm excitation occurs at a delay of 200 fs). Figure 5B shows the oscillator strength for the D_0_→D_2_ transition (*f*_02_) as a function of the C–N bond under the same torsional geometries as panel A. Here we can observe a sharp increase in *f*_02_ at an elongated C–N bond lenth of 1.54 Å (the equilibrium bond length is 1.46 Å for the optimized cation, Figure 3A). This increase is very large for the geometry with 87.4° dihedral angle but it is also visible, although to a lesser extent, for the geometry with dihedral angle of 52.7°. All this facts as a whole strengthen the assignment of the additional wave packet found in this work to the ring-stretching vibrational mode.

## 4 Discussion

This study represents the first demonstration of selective excitation to different electronic excited states upon coherent vibrational motion in a large organic cation. The motion of the two nuclear wave packets in PNT^+^ produces three distinct transient electronic transitions amenable to optical excitation: 1) D_0_→D_6_ transition with energy 3.1 eV at C–C–N–O dihedral angle of 0°, 2) D_0_→D_4_ (or D_3_) transition with energy 2.0 eV at a 90° dihedral angle, and 3) D_0_→D_2_ transition with energy 1.6 eV at 90° dihedral angle with elongated C–N bond length of 1.54 Å. The electronic structure of PNT^+^ along both the torsional and ring-stretching coordinates enables selective excitation to different excited states at specific geometries. The electronic structure was mainly explored by means of TDDFT calculations and further supported at the EOM-CCSD level. The upwards energetic shift observed for the D_2_, D_3_, and D_4_ states at the EOM level when compared with TDDFT results can be rationalized by the small 6−311+G* basis set being used for EOM computations (in contrast with the def2-TZVPP basis set for TDDFT calculations). Different groups had found that, as the basis set is expanded by adding diffuse and polarization functions, the excitation energies computed at the EOM-CCSD level decrease Cristian and Krylov (2003), Laurent et al. (2015). Therefore, a closer match between the excitation energies predicted by both levels of theory would be expected if a bigger basis set is employed for EOM calculations. Additionally, the discrepancy in which EOM identifies D_3_ as the bright state while TDDFT points to D_4_ has been previously observed for various systems Grimme and Parac (2003), Lopata et al. (2011), Prlj et al. (2015), Prlj et al. (2016), Acharya et al. (2018), López Peña et al. (2021) Nevertheless, both levels of theory confirm the presence of an excited state with substantial oscillator strength at 90° dihedral angle and with excitation energy around 2 eV. Overall, we conclude that the computationally inexpensive TDDFT level of theory is adequate enough for aiding and rationalizing the design of pump-probe control schemes.

**FIGURE 6:**
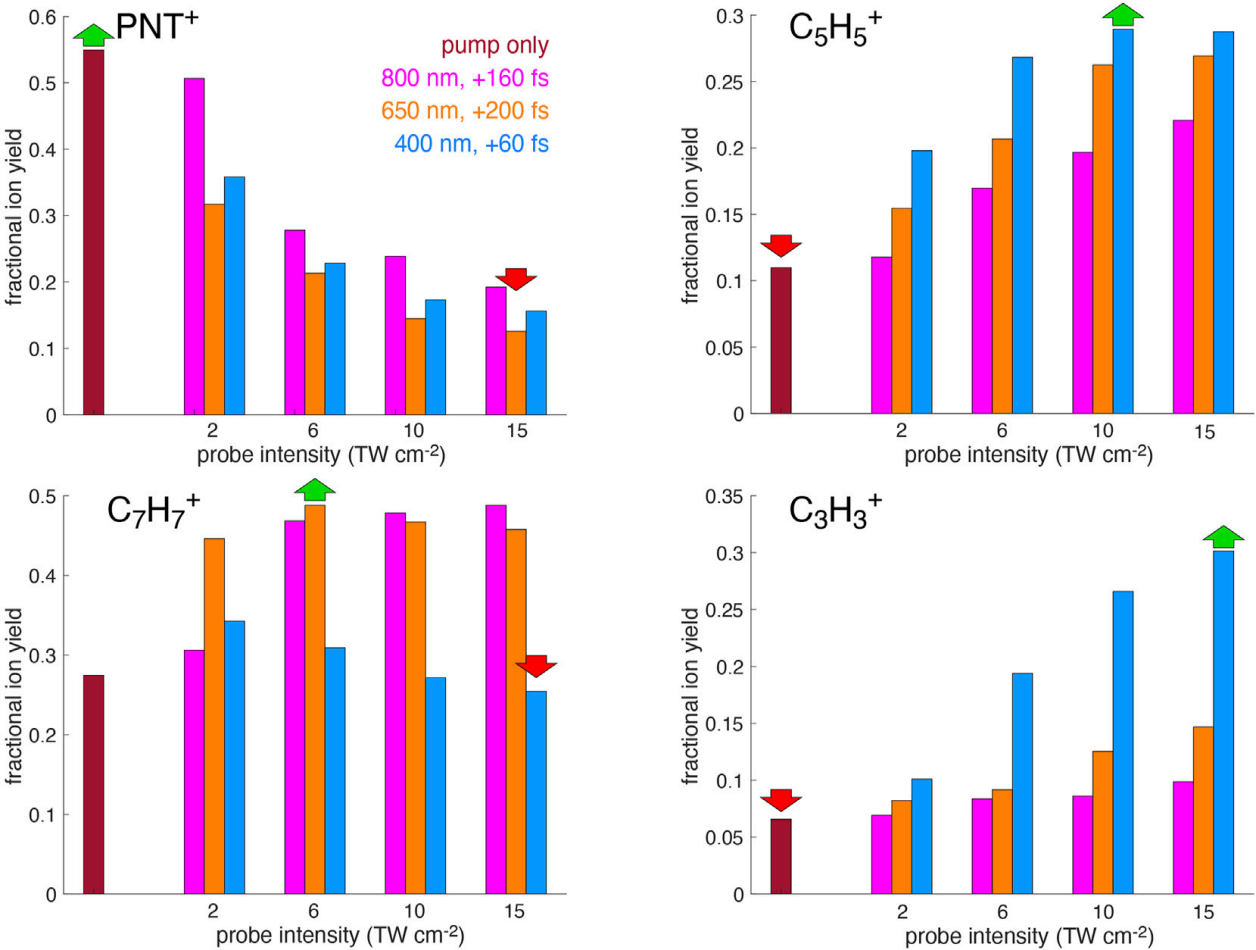
Fractional yields of PNT^+^, ${\text{C}}_{7}{{\text{H}}_{7}}^{+}$ , ${\text{C}}_{5}{{\text{H}}_{5}}^{+}$ , and ${\text{C}}_{3}{{\text{H}}_{3}}^{+}$ from Eq. 2 obtained using 800 nm, 650 nm, and 400 nm probe wavelengths at intensities from 2–15 TW cm^−2^.

The finding that 400 nm excitation of PNT^+^ selectively produces ${\text{C}}_{5}{{\text{H}}_{5}}^{+}$ and ${\text{C}}_{3}{{\text{H}}_{3}}^{+}$ , whereas 650 nm or 800 nm excitation produces ${\text{C}}_{7}{{\text{H}}_{7}}^{+}$ (Figure 1) indicates that population of the higher-energy D_6_ state causes more extensive fragmentation than population of the lower D_2_–D_4_ states. This finding is consistent with the reported higher dissociation energy of ${\text{C}}_{5}{{\text{H}}_{5}}^{+}$ and its formation by further dissociation of ${\text{C}}_{7}{{\text{H}}_{7}}^{+}$ Zhang et al. (2012). We can quantify the selectivity to ${\text{C}}_{7}{{\text{H}}_{7}}^{+}$ , ${\text{C}}_{5}{{\text{H}}_{5}}^{+}$ , or ${\text{C}}_{3}{{\text{H}}_{3}}^{+}$ formation by examining the fractional yield of a specific target ion relative to the sum of the ion yields,

$$Y=\frac{\text{target ion}}{{\text{PNT}}^{+}+{\text{C}}_{7}{{\text{H}}_{7}}^{+}+{\text{C}}_{5}{{\text{H}}_{5}}^{+}+{\text{C}}_{3}{{\text{H}}_{3}}^{+}}$$ (2)

for each probe wavelength at the pump-probe delay producing the greatest PNT^+^ signal depletion identified in Figure 1. The fractional yields for each ion at a series of probe intensities from 2 to 15 TW cm^−2^ are shown in Figure 6. Although greater enhancement of fragment ion yields is observed at higher probe intensities, 15 TW cm^−2^ represents a practical upper limit because at higher intensity the probe pulse creates ions even in the absence of the pump pulse. Examination of Figure 6 shows that ${\text{C}}_{7}{{\text{H}}_{7}}^{+}$ is selectively enhanced using 650 and 800 nm, whereas ${\text{C}}_{3}{{\text{H}}_{3}}^{+}$ is selectively enhanced using 400 nm. Hence, we can conclude that population of D_2_–D_4_ selectively produces ${\text{C}}_{7}{{\text{H}}_{7}}^{+}$ whereas population of D_6_ selectively produces ${\text{C}}_{3}{{\text{H}}_{3}}^{+}$ . Although the ${\text{C}}_{5}{{\text{H}}_{5}}^{+}$ yield is most enhanced by 400 nm excitation, its weaker dependence on the probe wavelength suggests that it can be formed by excitation to any of the excited states and is therefore less amenable to selective enhancement with pump-probe control. Overall, pump-probe excitation is found to enable a similar degree of control over the ${\text{C}}_{7}{{\text{H}}_{7}}^{+}$ / ${\text{C}}_{3}{{\text{H}}_{3}}^{+}$ ion ratios as attained using shaped 800 nm pulses Lozovoy et al. (2008): the maximum ${\text{C}}_{7}{{\text{H}}_{7}}^{+}$ / ${\text{C}}_{3}{{\text{H}}_{3}}^{+}$ ratio of 5.6 attained with 800 nm, 5 TW cm^−2^ probe pulses is 40% higher than the maximum ratio of ∼4 obtained with transform-limited 800 nm pulses, although the minimum ratio of 0.8 attained with 400 nm, 15 TW cm^−2^ is somewhat higher than the minimum ratio of ∼0.2 reported with pulse shaping.

Finally, the observation that strong field ionization of PNT launches nuclear wave packets along two distinct vibrational modes is of particular interest. Numerous previous pump-probe studies of other substituted benzenes including nitrobenzene López Peña et al. (2021), *o*-nitrotoluene Ampadu Boateng et al. (2019a), azobenzene Munkerup et al. (2017) and alkyl phenyl ketones Konar et al. (2014), Bohinski et al. (2014), Tibbetts et al. (2015) have observed only torsional wave packet motion upon strong-field ionization. To the best of our knowledge, the only previous study to definitively observe multimode coherent vibrational motion in a cation prepared by strong field ionization found a superposition of the C–I stretch and I–CH_3_ umbrella modes in CH_3_I^+^ Wei et al. (2017). Although in a previous work we had proposed multimode coherent excitation in diisopropyl methylphosphonate ion to explain observed ion yield oscillations at two different frequencies Ampadu Boateng et al. (2019b), no PES calculations were performed to confirm distinct excitation pathways. Hence, we recommend the combined strategy of pump-probe measurements with multiple probe wavelength and PES computations along possible coherently excited coordinates as performed in this work to identify possible multimode coherent excitation in other organic cations prepared by strong-field ionization.

## Data Availability

The original contributions presented in the study are included in the article/Supplementary Material, further inquiries can be directed to the corresponding author.

## Abbreviations

BBO, *β*-barium borate; DFT, density functional theory; EOM-EE-CCSD, equation-of-motion excitation-energies coupled cluster singles and doubles; OPA, optical parametric amplifier; PES, potential energy surface; PNT, *p*-nitrotoluene; TDDFT, time-dependent density functional theory; IVR, intramolecular vibrational energy redistribution.
