# Supplementary material for: Coherent Control of Molecular Dissociation by Selective Excitation of Nuclear Wave Packets
Source: Front Chem. 2022 Apr 5;10:859095. doi: 10.3389/fchem.2022.859095 (PMC9016217; doi:10.3389/fchem.2022.859095)
Supplement: Supplementary file 1 [file DataSheet1.pdf]

Supplementary Material

1 SUPPLEMENTARY TABLES AND FIGURES

1.1 Figures

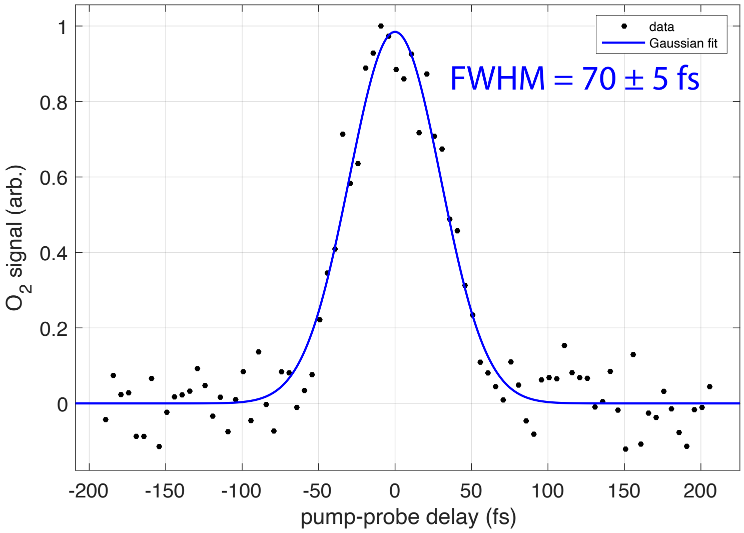

Figure S1. Ion signal of O_2 versus pump-probe delay with Gaussian fit. The 70 fs FWHM for the cross-correlation is taken as the width of the 400 nm probe pulse because the 1300 nm pump pulse has width of $\sim$20 fs.

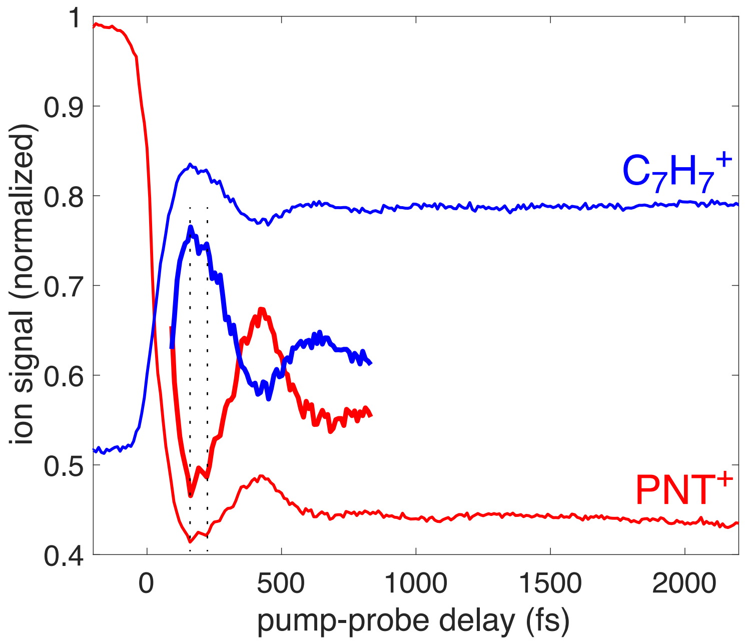

Figure S2. Transient ion signals from 1300 nm pump and 800 nm probe. The low-amplitude fast oscillations (highlighted by the magnification of ion signals and dotted lines) are less visible than using 1500 nm pump (Figures 1A and 2A).

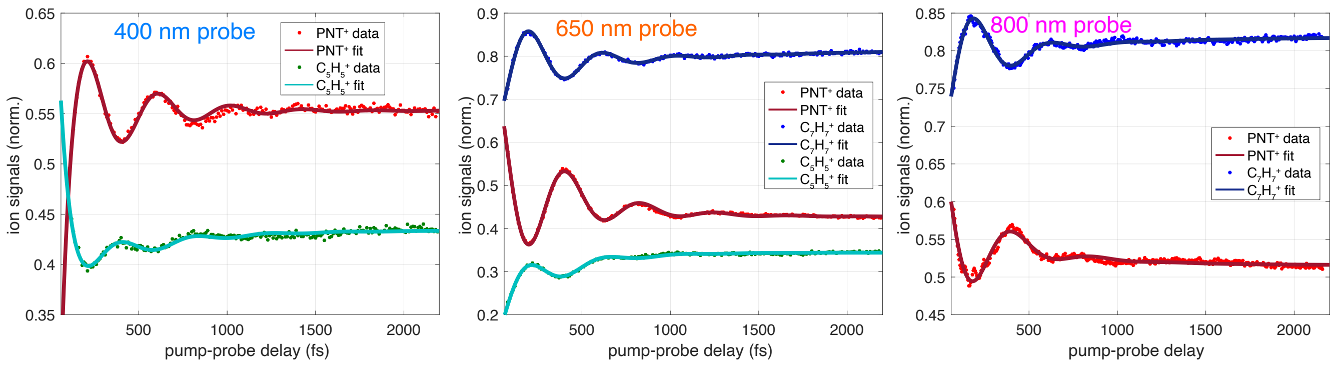

Figure S3. Transient ion signals and curve fitting to eq S1 or S2. Fit coefficients given in Tables S1–S3.

1.2 Tables

All transient signals were fit to the equation

$$S(\tau) = ae^{-\tau/T_1} \cos\left(\frac{2\pi}{t}\tau + \phi\right) + be^{-\tau/T_2} + c \tag{S1}$$

with the exception of the $C_5H_5^+$ signal for the 400 nm probe, which required one additional exponential decay term:

$$S(\tau) = ae^{-\tau/T_1} \cos\left(\frac{2\pi}{t}\tau + \phi\right) + be^{-\tau/T_2} + c + de^{-\tau/T_3} \tag{S2}$$

ion	a	T_1 (fs)	t (fs)	ϕ (rad)	b	T_2 (fs)	c	d	T_3 (fs)
PNT^+	0.10 ± 0.01	345 ± 33	410 ± 7	3.1 ± 0.1	-0.8 ± 0.6	32 ± 21	0.553 ± 0.001	-	-
$C_5H_5^+$	0.03 ± 0.01	370 ± 120	421 ± 20	0.3 ± 0.3	0.5 ± 0.3	56 ± 18	0.434 ± 0.001	-0.05 ± 0.01	470 ± 100

Table S1. Fit coefficients for 400 nm probe

.

ion	a	T_1 (fs)	t (fs)	ϕ (rad)	b	T_2 (fs)	c
PNT^+	0.25 ± 0.01	308 ± 15	425 ± 4	0.05 ± 0.04	0.1 ± 0.01	445 ± 49	0.427 ± 0.002
$C_7H_7^+$	0.15 ± 0.01	290 ± 12	417 ± 3	3.1 ± 0.03	-0.05 ± 0.01	2000 ± 700	0.83 ± 0.01
$C_5H_5^+$	0.09 ± 0.01	252 ± 17	438 ± 9	3.3 ± 0.1	-0.13 ± 0.01	316 ± 14	0.344 ± 0.001

Table S2. Fit coefficients for 650 nm probe

.

ion	a	T_1 (fs)	t (fs)	ϕ (rad)	b	T_2 (fs)	c
PNT^+	0.15 ± 0.02	210 ± 16	464 ± 11	0.45 ± 0.07	0.06 ± 0.01	476 ± 62	0.516 ± 0.001
$C_7H_7^+$	0.13 ± 0.01	212 ± 12	438 ± 7	3.3 ± 0.1	0.035 ± 0.002	682 ± 106	0.818 ± 0.001

Table S3. Fit coefficients for 800 nm probe

.

Element	X	Y	Z
C	0.041925	1.211232	-0.001089
C	-1.344135	1.200265	-0.005698
C	-2.060051	0.000000	-0.005278
C	-1.344135	-1.200265	-0.005698
C	0.041925	-1.211232	-0.001089
C	0.718977	0.000000	0.002393
H	-1.879673	2.140928	-0.012507
H	-1.879673	-2.140928	-0.012507
H	0.600068	2.134645	-0.003114
H	0.600068	-2.134645	-0.003114
N	2.191627	0.000000	0.004756
O	2.759942	1.081637	0.005917
O	2.759942	-1.081637	0.005917
C	-3.563730	0.000000	0.020795
H	-3.970858	0.883612	-0.469934
H	-3.931363	0.000000	1.050184
H	-3.970858	-0.883612	-0.469934

Table S4. Optimized geometric coordinates for neutral PNT

.

Element	X	Y	Z
C	0.035062	1.241740	-0.004135
C	-1.330460	1.233297	-0.014589
C	-2.055285	-0.000726	-0.017636
C	-1.331528	-1.235088	-0.007690
C	0.033997	-1.244191	-0.003338
C	0.703808	-0.001413	0.000405
H	-1.880933	2.164132	-0.020364
H	-1.882507	-2.165602	-0.011619
H	0.605342	2.159890	0.006811
H	0.603890	-2.162623	-0.010817
N	2.160385	-0.001909	0.009007
O	2.695131	0.657553	0.876098
O	2.702740	-0.662142	-0.853123
C	-3.528637	0.000282	-0.000898
H	-3.948916	0.896203	-0.456197
H	-3.861391	0.005353	1.051140
H	-3.950700	-0.897936	-0.449569

Table S5. Optimized geometric coordinates for PNT cation

.

Frequency (cm^{-1})	Intensity (a.u.)
11.06	1.98
58.96	1.31
79.68	0.58
164.31	1.22
215.71	4.76
302.93	1.40
336.83	3.45
367.52	2.13
372.00	1.73
514.15	14.06
565.45	0.28
604.32	5.20
689.17	2.88
741.16	13.70
775.10	1.18
809.57	1.56
822.64	18.64
852.58	31.74
984.91	76.45
992.00	3.15
1003.05	7.71
1021.56	0.36
1026.44	6.54
1139.26	14.17
1161.68	7.30
1218.77	52.84
1256.46	57.76
1283.82	3.40
1350.17	31.39
1360.23	128.69
1394.99	0.12
1411.79	13.61
1429.38	16.71
1478.83	146.47
1486.23	47.00
1494.06	43.95
1507.98	110.37
1657.12	89.10
2946.55	185.21
3075.05	2.47
3133.96	0.12
3201.83	0.93
3202.17	1.42
3217.16	27.27
3217.88	3.07

Table S6. Harmonic vibrational frequencies and intensities for optimized PNT cation

.

Element	X	Y	Z
C	-0.897345	0.686883	-1.770068
C	-1.533103	0.039917	-0.688463
C	-0.831542	-0.643580	0.327883
C	0.466437	0.645740	-1.828129
C	1.222476	-0.036716	-0.823855
C	0.531668	-0.676157	0.253261
C	2.695519	-0.055492	-0.881588
H	-1.486571	1.193360	-2.522060
H	-1.371594	-1.121723	1.133432
H	0.990699	1.128309	-2.641643
H	1.105340	-1.191331	1.011591
H	3.072678	0.036596	-1.899217
H	3.075095	0.821578	-0.330791
H	3.119074	-0.930298	-0.389807
N	-2.987829	0.082385	-0.615819
O	-3.574140	-0.793136	-1.213625
O	-3.464397	0.985419	0.035006

Table S7. Geometric coordinates for PNT cation with C–C–N–O dihedral angle of 87.4°

.

	B3LYP/Def2TZVPP		EOMCCSD/6-311+G*	
Transition	EE (eV)	f (a.u.)	EE (eV)	f (a.u.)
$D_0 \to D_1$	0.2223	0.0000	0.4563	0.000084
$D_0 \to D_2$	0.2375	0.0000	1.6238	0.000000
$D_0 \to D_3$	0.5579	0.0003	1.7845	0.001115
$D_0 \to D_4$	0.7020	0.0000	1.9550	0.000007
$D_0 \to D_5$	2.5512	0.0000	3.1075	0.000031
$D_0 \to D_6$	2.9536	0.0385	3.2137	0.064960
$D_0 \to D_7$	2.9625	0.0384	3.4620	0.000355

Table S8. Excitation energies (EE) and oscillator strengths (f) for PNT cation at the neutral optimized geometry (C–C–N–O dihedral angle of 0.1°) at the B3LYP/Def2TZVPP and EOMEE-CCSD/6-311+G* levels of theory

.

	B3LYP/Def2TZVPP		EOMCCSD/6-311+G*	
Transition	EE (eV)	f (a.u.)	EE (eV)	f (a.u.)
$D_0 \to D_1$	0.9244	0.0000	1.0542	0.000072
$D_0 \to D_2$	1.3207	0.0009	2.2312	0.001051
$D_0 \to D_3$	1.6492	0.0000	2.3223	0.083023
$D_0 \to D_4$	1.7062	0.0811	2.4925	0.000173
$D_0 \to D_5$	3.0109	0.0002	3.4981	0.000631
$D_0 \to D_6$	3.3806	0.0116	3.6250	0.006788
$D_0 \to D_7$	3.6314	0.0004	3.6558	0.000170

Table S9. Excitation energies (EE) and oscillator strengths (f) for optimized PNT cation (C–C–N–O dihedral angle of 52.7°) at the B3LYP/Def2TZVPP and EOMEE- CCSD/6-311+G* levels of theory

.
